# Regulatory T-Cells and Multiple Myeloma: Implications in Tumor Immune Biology and Treatment

**DOI:** 10.3390/jcm10194588

**Published:** 2021-10-05

**Authors:** Christina Hadjiaggelidou, Eirini Katodritou

**Affiliations:** Department of Hematology, Theagenio Cancer Hospital, 54639 Thessaloniki, Greece; eirinikatodritou@gmail.com

**Keywords:** multiple myeloma, regulatory T-cells, tumor microenvironment

## Abstract

Multiple myeloma (MM) is associated with both cellular and humoral immune deficiencies and, despite significant advances in treatment, remains an incurable disease. Regulatory T-cells (Tregs) represent a critical subset of CD4 T-cells, characterized by CD4 + CD25+ Forkhead box P3+ (FoxP3+) phenotype, able to control peripheral tolerance and responses to foreign and tumor antigens. Tregs are elevated in various types of cancer, including hematological malignancies; in MM, data regarding Tregs function and numbers and their correlation with survival parameters are controversial. Advances in cancer biology have shown that the tumor microenvironment plays an important role in tumor progression. In MM, the highly immunosuppressive nature of the bone marrow microenvironment has been significantly elucidated in the past decade and it is now well acknowledged that targeting only the tumor clone may not be able to cure MM. Tregs within the tumor microenvironment might play a significant role in the suppression of antitumor immune responses against cancer cells and are considered to predict poor outcome in cancer patients; nonetheless the exact prognostic significance of this cell subpopulation in malignancies is still a matter of debate. In this review, we discuss the role of Tregs as an essential cell population of the MM immune microenvironment.

## 1. Introduction

Multiple myeloma (ΜΜ) is a hematopoietic malignancy characterized by the proliferation of plasma cells within bone marrow leading to anemia, bone destruction, hypercalcemia, renal failure, and infections. It accounts for ≈10% of hematological malignancies and has an overall annual incidence of 4.4 cases per 100,000 population [1]. Despite survival improvements attributed to the wide use of autologous peripheral stem cell transplantation (ASCT) and the introduction of novel antimyeloma agents, i.e., proteasome inhibitors, immunomodulating drugs (IMiDs), and recently monoclonal antibodies, MM remains an incurable disease [1]. The major pathogenic mechanisms include primary and secondary translocations, defects of cyclins and cell cycle regulators, dysregulation of signal transduction pathways, and interactions between stromal and malignant plasma cells [2]. The pathological interactions between myeloma plasma cells and the bone marrow microenvironment create an immunosuppressive network that is vital for the maintenance and progression of the disease [3,4,5]. Multiple myeloma has been repeatedly associated with reduced immune surveillance concerning abnormalities in number and function of B-cells as well as substantial impairment of the cellular immune system including abnormalities in T, natural killer (NK), NK-like cells (NKL) and dendritic populations [6,7]. Immune dysfunction can facilitate severe infectious complications or compromise immunotherapeutic interventions which can both cause major morbidity and mortality [6]. Regulatory T-cells (Tregs) constitute a small-size subpopulation of circulating CD4 + T-cells (1–4%), which play a central role in maintaining immunological unresponsiveness to self-antigens and in suppressing unwanted immune responses toward foreign antigens in the context of immunological tolerance [8,9,10]. In the past decade, Tregs have attracted great attention due to their ability to deteriorate immune responses against a wide panel of neoplasms, including MM [8,9,10]. Most of the studies have shown that Tregs are elevated in various types of cancer (e.g., lung, ovarian, liver, pancreatic, breast cancers, melanoma), including hematological malignancies (e.g., lymphoproliferative disorders, Hodgkin disease) and can eliminate protective antitumor immunity, contributing on tumors’ progression [11,12,13,14,15,16,17,18,19,20,21,22]. In MM, reports concerning Tregs numbers and function are conflicting [10]. In addition, data regarding the impact of current antimyeloma therapies, including autologous stem cell transplantation (ASCT) and novel agents i.e., proteasome inhibitors, IMiDs and monoclonal antibodies (MoAbs) on Tregs number and function are also contradictory. Among novel agents, IMiDs and CD38 MoAbs seem to improve host-antitumor immunity, by the elimination of Tregs whereas, the combination of those drugs, can possibly lead to further enhancement of the immune response, relieving eventually the immunosuppressive bone marrow microenvironment. In this review, we discuss the role of Tregs as an essential cell population of MM immune microenvironment and their implication on myeloma therapy. 

## 2. Tregs: Properties and Function

Immunological tolerance refers to several immune functions which lead to maintaining a state of unresponsiveness to autoantigens while generating protective immunity against invading pathogens [23]. The immune system uses various mechanisms to maintain immunologic self-tolerance and protect the host against exacerbated responses to foreign antigens. The hypothesis of the existence of regulatory T-cells, originally termed suppressor T-cells, was first supported in the early 1970s [24], when Gershon and Kondo proposed that a subpopulation of CD8+ T-cells exhibited suppressive activity [25,26]. However, it was not only until 1995, when a landmark study by Sakaguchi and colleagues described a unique CD4 + CD25 + T population with potent regulatory activity [26,27]. Tregs represent a critical subset of CD4 T-cells, characterized by CD4 + CD25 + Forkhead box P3+ (FoxP3+) phenotype, able to control peripheral tolerance as well as response to foreign and tumor antigens [8,9,10,26]. High expression of the high affinity IL-2 receptor α chain, CD25 was first identified in a small subset of T-cells with regulatory properties by Baecher-Allan et al. [28], while intracellular Forkhead family transcription factor FoxP3 was reported in 2003 as a critical regulator of Treg development, function and homeostasis [29,30,31]. The importance of FoxP3 as a crucial transcription marker became apparent in situations of single gene mutation on the X chromosome which led to severe autoimmune/inflammatory diseases in both Scurfy mice and humans [32,33]. In particular, patients with mutations in FoxP3 developed a severe, fatal systemic autoimmune disorder called Immune dysregulation Polyendocrinopathy Enteropathy X-linked (IPEX) syndrome characterized by autoimmune manifestations in multiple endocrine organs, such as diabetes and thyroiditis, inflammatory bowel disease, and severe allergies [32,33]. Finally, low or negative expression of the heterodimeric IL-7 receptor (CD127) combined with high expression of CD25 on CD4 + FoxP3+ cells characterize regulatory cells with mainly immunosuppressive properties and represent 1–2% of the total number of Tregs [34,35]. Tregs can be broadly subdivided in two main subpopulations which have different origin of development: naïve Tregs (nTregs) and induced Tregs (iTregs). Specifically, nTregs are generated from progenitor cells in the bone marrow and develop in thymus in the context of positive and negative selection while iTregs are induced in the periphery from naïve T-cells and are subdivided in type 1 regulatory T-cells (Tr1) and in T helper 3 (Th3) cells which acquire regulatory properties via IL-10 and TGF-β respectively [8,9,10]. Another widely used classification of human Tregs is based on the expression of CD45RA+ (naïve-like or resting Tregs) and CD45RO+ (activated effector or memory-like Tregs) [10,36]. Tregs can be classified into three fractions using FoxP3, CD25, and CD45RA: 1) fraction I (Fr. I) CD45RA + FoxP3^low^/CD25^low^ resting or naïve Tregs, 2) Fr. II CD45RA−FoxP3^high^/CD25^high^ effector Tregs and 3) Fr. III CD45RA−FoxP3^low^/CD25 ^low^ cells, the majority of which are not Tregs [36,37]. This classification indicates that FoxP3 + T-cell represent a heterogeneous population including naïve and effector Tregs and non-Tregs, with different functional and phenotypic characteristics.

Other lymphocyte populations with regulatory properties such as gamma delta (γδ) T-cells, CD8 + T-cells, double negative cells, and regulatory-B-cells (Bregs), do not always express CD4. Gamma delta T-cells comprise a small subset of T-cells that are distinguished by their γδ-T-cell receptors (TCR) and are mostly found in tissues and tumor sites where they exert their suppressive activity towards naïve and effector T-cell responses; they also impede proliferation and function of dendritic cells (DC) [38,39,40,41]. Bisphosphonates and IL-2 are both known to be potent γδ T-cell stimulators [7]. Double negative cells (alpha beta TCR + CD3 + CD4-CD8-) disseminate in the peripheral blood and can inhibit immune responses in an antigen-specific fashion [42,43,44]. Several subsets of CD8 can also display regulatory activity under certain circumstances (i.e., Qa-1 restricted CD8 + Tregs, CD8 + CD28-Tregs, CD8 + CD25^high^ Tregs); most of these cells become regulatory in an antigen-specific manner when stimulated by alloantigens and antigens presented via plasmacytoid DC [45,46,47,48,49,50]. Bregs, on the other hand, represent certain B lymphocyte subsets which have immunosuppressive properties and contribute to immunological tolerance; their activity is rather the result of their dynamic interaction with other cells of the immune system [51,52].

Although there is no molecular marker that uniquely identifies Tregs, there are several factors and cell surface proteins with diverse expression on Tregs such as FoxP3, CD25, cytotoxic T-lymphocyte-associated protein 4 (CTLA-4) and CD127, which contribute accordingly in a different manner to their regulatory properties [9,53]. Furthermore, various cytokines including interleukins 2, 6, 17 (IL-2, IL-6, IL-17), and tumor growth factor β (TGF-β) can influence the number and activity of Tregs, negatively or positively, in physiologic and pathologic conditions [54]. In particular, ΙL-2 reflects CD25 alpha chain receptor and promotes FoxP3 expression in Tregs in vivo and in vitro. It is also essential for the induction of iTregs and for maintaining Tregs activity in vivo [9,10]. Tumor growth factor β plays a major role in Tregs differentiation, proliferation, and suppressive functions and its co-existence with ΙL-2 is required for the conversion of nTregs to iTregs [8,9,55]. Interleukin-17 is produced by Th17 cells which are related to Tregs in a reciprocal way [56] and differentiate from naïve CD4 cells in the presence of IL-6 with or without TGF-β [57,58]. Finally, inhibitory cytokines IL-10, and IL-35 expressed by Tregs are implicated in their major mechanisms of suppression [9].

How Tregs exert their immunosuppressive activity has been analyzed and discussed in numerous publications during recent decades [9,27,59,60,61,62]; taking together published data, it has become evident that Tregs do not rely on a single mechanism of suppression but rather have an arsenal of regulatory mechanisms at their disposal [9]. In general, human CD4 + CD25 + FoxP3 + Tregs can exert suppressive function in four distinct ways: (1) release of inhibitory cytokines such as IL-10, IL-35, TGF-β, (2) mediation of cytolysis through granzyme A/B and perforin, (3) metabolic disruption of the target cell and (4) modulation of antigen-presenting cells (APCs) and dendritic cells (DCs) function [9,33,34] via interaction of cell surface molecules on Tregs such as CTLA-4 and the lymphocyte activation gene 3 (LAG-3) with CD80/CD86 and MHC class II respectively, on APCs [9,27,59,60,61,62,63,64,65]. This interaction results in the reduced ability of the APCs to activate conventional T-cells. Furthermore, there is evidence that Tregs promote the production of the immunoregulatory tryptophan-degrading enzyme, indoleamine 2,3-dioxygenase (IDO) by DCs [66].

## 3. Tregs and Tumor Microenvironment

Tregs have a fundamental role in immune homeostasis in healthy individuals as they are responsible for maintaining a state of unresponsiveness to autoantigens on one hand and on the other hand, they develop protective immunity against invading pathogens [23]. In the context of tumor immunology, immunity, or immune tolerance means the success or failure, respectively, of the immune system to reject a tumor. The tumor microenvironment, which is composed of immune cells, stromal cells, and the extracellular matrix surrounding tumor cells, is a main battleground during the neoplastic process, fostering proliferation survival and migration of tumor cells [5]. Furthermore, an array of cytokines and immune-modulating agents have been reported by many investigators in the field of tumor immunobiology [67]. Published data show that while Tregs are critical for the peripheral maintenance of potential autoreactive T-cells, they can be detrimental as they negatively affect effective antitumor responses [8]. Tregs are recruited and accumulated in the tumor microenvironment via mechanisms partially elucidated, where they play a significant role in the suppression of antitumor immune responses against cancer cells [11,12]. In normal conditions, Tregs migration process from thymus to the peripheral blood and to secondary lymphoid organs encounters several chemokine receptors (i.e., CCR2, CCR4, CCR6, CCR7, CCR8 and CCR9) with chemotactic response to certain ligands (i.e., CCL22 and CCL17) [10]. In tumor microenvironment, changes in receptors’ expression and chemokines’ function mediate the suppressive activity of Tregs. The main mechanisms used by Tregs to eliminate protective antitumor immunity contributing on tumor progression, include direct inhibition of effector T-cells, dysfunction of DCs via IL-10 and TGF-β and interruption of CD4 T-cell-mediated generation of CD8 T-cell cytotoxic responses. Furthermore, molecules such as CTLA-4 and the inducible T-cell co-stimulator (ICOS) expressed by Tregs are involved in their suppressive activity. Overall, most studies report that Tregs are elevated in various types of cancer (e.g., lung, ovarian, liver, pancreatic, breast cancers, melanoma), including hematological malignancies (e.g., lymphoproliferative disorders, Hodgkin disease) and can eliminate effective antitumor activity, contributing to progression of malignant disease [11,12,68,69,70]. However, there are few reports which have demonstrated that higher numbers of Tregs in tumors are associated with a better prognosis [71,72,73]. Moreover, few studies have reached the conclusion that high numbers of circulating CD4 + CD25 + FoxP3+ cells are associated with reduced incidence of graft versus host disease (GVHD) after allogeneic stem cell transplantation [74,75]. Taken together, Tregs within the tumor microenvironment might play a significant role in the suppression of antitumor immune responses against cancer cells and are considered to predict poor outcome in cancer patients; nonetheless the exact prognostic significance of this cell subpopulation in malignancies is still a matter of debate [26].

## 4. Tregs and Their Role in Multiple Myeloma

Functionality and Frequencies of Tregs in Myeloma Immune Microenvironment

Multiple myeloma originates from asymptomatic precursor conditions, specifically monoclonal gammopathy of undetermined significance (MGUS) and smoldering MM (SMM) and is associated with both cellular and humoral immune deficiencies [76,77]. The process of transformation of MGUS to symptomatic MM is related to sequential genetic mutations but also with significant changes in the cellular composition of the bone marrow microenvironment [7,77,78,79,80]. Ιmmune impairment is an important feature of MM, concerning both humoral and cellular immune system and consists of decreased levels of uninvolved immunoglobulins and dysfunctional T-cell responses, correlating with increased morbidity and mortality [2,6,7]. The relationship between myeloma plasma cells and the bone marrow microenvironment is critical for the maintenance of disease and the subsequent loss of functional immune surveillance [3,4]. The interplay between tumor and stromal cells is accomplished via adhesion molecules and cytokines networks and consistently promotes tumor cell survival, drug resistance, angiogenesis, and disordered bone metabolism [40]. In addition, significant reductions in the numbers of CD4, CD8 T and CD19 B populations combined with increased levels of several immunologically active compounds such as TGF-β, vascular endothelial growth factor (VEGF), IL-10 and IL-6 have been shown to correlate with increased morbidity and mortality indicating a potential positive relationship between cellular components of immune system and disease control [2,6,7,40]. Cellular changes in the bone marrow microenvironment during myelomagenesis involve the development and/or recruitment of various immunosuppressive cells, including myeloid-derived suppressor cells (MDSCs), tumor-associated macrophages (TAMs), regulatory B-cells (Bregs) and regulatory T-cells (Tregs). Myeloid-derived suppressor cells (MDSCs) are activated immature myeloid cells that lack the expression of mature lymphoid and myeloid markers as well as human leukocyte antigen HLA-DR (major histocompatibility complex MHC class II) and accumulate in the tumor environment where they exert their immunosuppressive activity [10]. According to published data, MDSCs may contribute to MM progression, as they can induce Tregs differentiation, suppress T-cell proliferation, promote angiogenesis and proliferation of myeloma cells, and even differentiate themselves into functional osteoclasts [81].

Studies of Tregs in the bone marrow of patients with MM are relatively few compared to those concerning peripheral blood. Published reports show that Tregs frequencies in both departments are similar [82,83,84]. Nevertheless, there is evidence that increased numbers of Tregs in the bone marrow correlate with adverse clinical features, such as hypercalcemia, lower normal plasma cell count and IgA myeloma subtype [84]. Another study showed that FoxP3 and CTLA4 overexpression in bone marrow mononuclear cells of MM patients was a sign of Tregs accumulation in that compartment [85]. Even though Tregs have been reported to be expanded and functional in a variety of cancers, published data regarding Tregs numbers and function in MM are conflicting [86]. Various groups have shown increased frequency of functional Tregs in the peripheral blood of MM patients compared to those of healthy donors [44,83,84,87,88] and most of the studies suggest that Tregs are as suppressive against conventional T-cell populations in myeloma patients as in healthy subjects [44,83,87,89]. Beyer et al. reported strong inhibitory function of Tregs in peripheral blood of MM patients and confirmed that they express increased level of TGF-β and IL-10 when compared to healthy subjects [83]. High concentrations of Tregs-associated markers, such as CTLA-4, glucocorticoid-induced tumor necrosis factor receptor (GITR) and OX40 are reported in peripheral blood of MM patients, and this might enhance their suppressive function [83]. Cytotoxic T-lymphocyte-associated protein-4 (CTLA-4) along with PD-1/PD-L1 represent two major immune checkpoints in MM responsible for maintaining immune tolerance and controlling the duration and intensity of immune responses [38,90,91]. Published studies consider FOXP3 and CTLA4 overexpression in MM bone marrow as a sign of accumulation of Tregs and claim that CTLA4 + Tregs are increased in the bone marrow of patients with MM compared with patients with MGUS and with healthy donors [85,92,93,94]. The programmed cell death protein PD-1/PD-ligand 1 pathway plays a critical role in the balance between T-cell activation and tolerance and regulates the dynamic interplay between Tregs and T-effector cells [95]. Moreover, γδ-T-cells isolated from myeloma patients overexpress PD-1 which may be related to decreased function of effector cells [96,97].

Cytokines such as TGF-beta and IL-10 are increased in the myeloma BM, reflecting the immune suppressive microenvironment [44]. In addition, other cytokines related to myeloma biology, including interleukins 2, 6, 17 (IL-2, IL-6, IL-17) can modulate Τregs function [38,98]. Inhibitory cytokines (TGF-beta, IL-10 and IL-35) and cytolytic granules (granzymes, perforins) are used by functional Tregs to exert their suppressive activity [59]. Various reports demonstrate that Tregs enable inhibiting the proliferation of CD4+ T-cells and the secretion of IFN-γ [26,35,90]. Similarly, functional studies by Foglietta et al. [99] and Arena et al. [100] showed effective suppressor activity in myeloma patients. On the other hand, Prabhala et al. report that Tregs in MM lack suppressive activity due to their reduced ability to inhibit anti-CD3-induced proliferation [101]. These opposing results might be due to differences in assay and purification techniques. For example, Prabhala et al. [101] used whole-blood mononuclear cells for the stimulation experiments, while Beyer at al [83] used purified CD4+ cells. The disagreement in the literature does concern not only myeloma Tregs function, but also their frequencies. There are conflicting reports of Tregs frequency being increased [44,83,84,88], decreased [89,101], and unchanged [99,100] in myeloma patients as compared with controls [102]. This discrepancy may be likely explained by the heterogeneity of samples that have been studied (i.e., whole-blood compartment, peripheral-blood mononuclear cells, bone marrow), the variety of identification processes applied throughout the aforementioned studies and the fact that there is no consistency on how Tregs numbers are reported (either % frequencies or absolute values) [26]. The precise gating strategies of Tregs are still under debate, therefore different groups have been using different markers for Tregs quantification which may lead to different results regarding Tregs frequencies in MM patients [82]. More specifically, Feyler et al. [44] identified peripheral-blood Τregs as CD4 + CD25 + FoxP3+ cells while Beyler et al. [83] and Giannopoulos et al. [88] took into consideration the intensity of CD25 expression and identified Tregs as CD4 + CD25^high^FoxP3+ cells. Brimnes et al. [87] found increased levels of CD4 + FOXP3 + Tregs at diagnosis but not in patients in remission or with MGUS. Muthu Raja et al. as well (84), reported increased frequencies of CD4 + CD25 + CD127^low/dim^ FoxP3 + cells in MM patients, but not in those with SMM and MGUS. Interestingly, DCs and MDSCs decreased in the same group of patients. In our study [103], we identified Tregs as CD4 + CD25^high^CD127^low/dim^ FoxP3+ cells based on literature data according to which high expression of CD25 combined with low or negative expression of CD127 on CD4 + FoxP3+ cells characterize regulatory cells with mainly immunosuppressive properties and represent 1–2% of the total number of Tregs [53,104]. In cases of reported decreased Tregs in untreated myeloma patients compared with healthy subjects, Prabhala et al. [101] identified Tregs as CD4 + FoxP3+ and Gupta et al. [89] characterized Tregs with the inclusion of CD127 in gating. Data regarding Tregs correlation with survival parameters and disease outcome are very limited and need further evaluation. Giannopoulos et al. have shown that patients treated upfront with thalidomide triplets, who had high Tregs frequencies, displayed significantly reduced overall survival compared with patients with reduced Tregs [88]. Muthu Raja et al. reported that patients with higher levels of Tregs in the peripheral blood had shorter time to progression compared to patients with lower levels [105]. According to Feyler et al., Tregs numbers correlated with paraprotein levels and disease burden; particularly relatively higher numbers of Tregs were observed in myeloma patients with stage I and II according to the International Staging System (ISS) and low disease burden as compared to those with relapsed or refractory disease [44]. Finally, decreased number of Tregs correlated with ISS II and III in Gupta’s study [89]. In our study, Tregs% reduction between baseline and response marginally correlated with PFS, in patients treated with lenalidomide in combination with dexamethasone in early lines, in the univariate cox regression analysis; however, this observation could not be validated in a multivariate cox regression model due to the limited number of patients [103].

Regarding the pro-inflammatory Th17 cells, the Treg/Th17 balance is considered to be an immunoregulatory marker [7]. Published studies demonstrate a reciprocal relationship between Tregs and Th17 cells in the context of MM, which is further supported by the presence of IL-6 [56,57,58]. The amount of Th17 cells in the BM positively correlates with clinicopathological characteristics in MM, such as clinical tumor stage, serum lactate dehydrogenase concentration, and serum creatinine concentration [106]. MM cells skew the Treg/Th17 balance to induce an immunosuppressive state [107] and long-term survival in MM is associated with a favorable Treg/Th17 balance [58,106,108,109]. Furthermore, Noonan et al. demonstrated that the Th17 T-cell phenotype may serve as a key predictor of lytic bone disease in multiple myeloma [58]. In the previously mentioned studies, MM patients had increased levels of IL-17 compared to controls; this could be related to the increased amounts of IL-6 in the bone marrow of myeloma patients with active disease which promotes the production of Th17 cells from CD4 naïve cells.

## 5. Tregs Correlations with Myeloma Therapy

The wide use of autologous peripheral stem cell transplantation (ASCT) and the introduction of novel myeloma therapies [1] have substantially improved survival in myeloma patients. Although the immune system has been implicated in the development of MM, data regarding the role of various immune components in this process are broad and sometimes contradictory. Current treatment options such as proteasome inhibitors, IMiDs, MoAbs, ASCT and bisphosphonate’s support, promote immune reconstitution and eliminate the bone marrow microenvironment mediated immune evasion in coordination with the individual patient’s profile, including cytogenetics and molecular signature [110]. 

Data concerning Tregs alterations during therapy with IMiDs and proteasome inhibitors are limited and controversial. Giannopoulos et al. [88] demonstrated that Tregs frequencies in myeloma patients treated with thalidomide triplets and in some cases followed by ASCT, either remained stable or were significantly increased. In addition, patients who progressed and did not respond to therapy demonstrated the highest values of Tregs. Galustian et al. [34] reported that the expansion of CD4 + CD25 + ^high^CTLA4 + Foxp3+ Tregs which were isolated from peripheral-blood mononuclear cells (PBMCs) treated with IL-2 decreased after incubation with lenalidomide, and this was mainly attributed to the suppression of transcription factors FoxP3 and OX40 (CD134). According to Galustian et al., lenalidomide and pomalidomide may enhance antitumor immunity by inhibiting the suppressive effects of regulatory cells [34]. On the contrary, Muthu Raja et al. [35] reported that lenalidomide in combination with dexamethasone increased Τregs numbers in newly diagnosed patients and this could negatively influence the antitumor immune response, according to authors speculations. In our study, [103], Tregs significantly reduced after treatment with lenalidomide and dexamethasone; the opposite results demonstrated in our study compared to the study of Muthu Raja [35] could be attributed to the difference of the quality of response to therapy, correlating complete response and very good partial response with significant decrease in Tregs frequencies. Data concerning Tregs alterations during therapy with bortezomib and next generation proteasome inhibitors such as carfilzomib are very limited. Blanco et al. [111] reported that the addition of bortezomib to CD4 T-cells cultures not only does not affect the viability of nTregs, but furthermore it promotes the emergence of a distinct suppressor CD4 T-cell population while eliminating the activities of conventional T-cells. Interestingly, a few studies have reached the conclusion that high numbers of circulating CD4 + CD25 + FoxP3+ cells are associated with reduced incidence of graft versus host disease (GVHD) after allogeneic stem cell transplantation [74,75]. According to Blanco et al., resistance of Tregs to the pro-apoptotic effect of bortezomib could be used as a potential therapeutic tool against GVHD [111]. In our study, bortezomib-based treatment had no impact on Tregs numbers, underscoring the different mode of action of proteasome inhibitors [105].

Daratumumab is the first human immunoglobulin G1 (IgG1) MoAb that targets CD38 approved for use in MM with significant clinical activity in relapsed and refractory MM [112,113,114] and recently for the treatment of newly diagnosed MM patients, in combination with other novel agents such as bortezomib, thalidomide and dexamethasone for transplant eligible patients, or with lenalidomide and dexamethasone for elderly symptomatic MM patients [115]. Immunosuppressive populations such as MDSCs and Bregs which contribute to tumor growth and promote immune evasion, express CD38 and can be susceptible to daratumumab treatment [112,116,117]. Interestingly, a novel subpopulation of Tregs (CD4 + CD25 + CD127^dim^) was identified that also expressed high levels of CD38 and demonstrated superior autologous T-cell suppressive capacities. These cells were also sensitive to daratumumab and were significantly reduced in patients receiving treatment [118,119]. Daratumumab-mediated elimination of these CD38+ immune regulatory cells may reduce local immune suppression within the myeloma microenvironment and restore immune effector function against the disease [120,121]. In addition, CD38 is a multifunctional ectoenzyme which exhibits a NADase activity which contributes to the development of T-cell exhaustion via reducing nicotinamide adenine dinucleotide (NAD+) levels. Interestingly, CD38 inhibition on T-cells by CD38 MoAbs improves antitumor activity by increasing NAD+ levels [122,123]. Another CD38 MoAb which has proved to exhibit antimyeloma activity in clinical studies, in the relapsed/refractory MM setting is Isatuximab [115,120]. Isatuximab can preferentially block immunosuppressive Tregs which highly express CD38, by decreasing their percentages, reducing immune inhibitory cytokines (TGF-β, IL-10), and blocking their trafficking allowing thus positive immune effector cells to expand and contribute to antitumor response [120,124]. Of note, according to published data IMiDs, such as lenalidomide and pomalidomide may enhance the expression of cell surface CD38 on Tregs of patients with MM, conferring further sensitivity to CD38 MoAbs treatment [118]; moreover, the concomitant administration of MoAbs that target CD38 with IMiDs further enhances NK- and CD8+ T-effector cell-mediated antitumor immune responses and antibody-dependent cell-mediated cytotoxicity (ADCC), strengthening thus antimyeloma activity [120]. Taking into consideration the important contribution of triggering antimyeloma immunity in disease control, we believe that the combination of anti-CD38 MoAbs with IMiDs seems to be an extremely attractive therapeutic approach, for the treatment not only of active MM, but also of smoldering MM (SMM); studies examining the efficacy of CD38 MoAbs in combination with lenalidomide for high-risk SMM are ongoing [125]. The impact of conventional drugs on immune response has been examined in a study by Muthu Raja et al. which demonstrated that CD4 T-cells along with Tregs are reduced after treatment with cyclophopshamide, in combination with thalidomide, and dexamethasone (CDT) in patients that achieved ≥vgPR, probably because of the cytotoxic effect of cyclophosphamide [107]. Finally, apart from the impact of IMiDs, proteasome inhibitors and MoAbs on immune cell responses, several reports support that bisphosphonates may activate γδ-T-cells in exhibiting cytotoxic activity against myeloma cells [126,127,128,129]. Despite beneficial immunomodulatory properties of novel drugs, particularly of IMiDs and MoAbs, which facilitate a long-lasting tumor control in MM, both agents may increase the risk of infections during the initial phase of MM treatment or at relapse; The major impact of those agents is the exacerbation of lymphopenia which could result to severe opportunistic infections. [6,130]; In addition, proteasome inhibitors increase the risk of viral infections such as varicella zoster mainly via depletion of B-cells. Administration of chemoprophylaxis as well as vaccinations is warranted to minimize the risk of severe and potentially lethal infectious complications [6,130].

## 6. Future Perspectives

Recent advances in cancer biology have shown that not only the tumor cell themselves but also cells that constitute immune microenvironment have a significant role in oncogenesis and tumor progression. In MM, the highly immunosuppressive nature of the bone marrow microenvironment and its contribution in myeloma cell development and survival, has been significantly elucidated in the past decade and it is now well acknowledged that targeting only the tumor clone may not be able to cure MM. Therefore, it is vital to develop novel therapeutic agents that not only eliminate the tumor clone itself but also target the disease immune microenvironment to modulate effectively important immune escape mechanisms [82].

The efficacy of novel immunotargeting therapy in MM, such as MoAbs targeting B-cell maturation antigen (BCMA), checkpoint inhibitors, and chimeric antigen receptor (CAR) T-cells, either as monotherapy or in combination with other novel myeloma agents is currently assessed in several ongoing MM clinical trials [131,132,133,134,135,136]; however, there is no data, regarding the impact of those therapies on Tregs homeostasis and function in MM. On the other hand, the essential role of CD4 + CD25 + FoxP3+ regulatory T-cells in the control of physiological as well as pathological immunity is now well established, albeit many aspects of their biology yet, remain unclear [8]. The enhanced expansion and suppressive activity of Tregs contributing to tumor cell growth, proliferation, and survival, has been elucidated in both solid and hematological malignancies including MM [26,28,34,68,137,138]. Therefore, depleting Tregs and inhibiting their suppressive effects via targeted therapy as well as by chemo-radio-therapeutic modalities could be a useful strategy to intensify antitumor immunity [139]. The perspective on the realization of Treg depleting or inhibiting therapies in the clinic is currently under investigation [26,37,140]. Treg-down strategies include targeting cell surface molecules selectively expressed on tumor-infiltrating Tregs such as CD25, CTLA-4, GITR, 4–1BB, OX-40, LAG3, and T-cell immunoglobulin and ITIM domain (TIGIT), and some c“The authors declare no conflict of interest.” hemokine receptors such as CCR4 and CCR8 withies monoclonal antibodies [141,142,143,144,145] or using small molecules such as imatinib or PI3Kδ, c-Rel and CARMA1 to selectively target effector Tregs [146,147,148,149]. Overall, in MM, published data regarding Tregs number and function are controversial and this disagreement in the literature is most likely explained by the heterogeneity of the experimental approaches that are used [26]. In addition, information concerning Tregs alterations during treatment with currently available myeloma agents and possible correlations with survival parameters are limited and require further investigation. However, there is strong evidence that MoAbs targeting CD38 in combination with IMiDs suppress the inhibitory function of Tregs which highly express CD38 and enhance NK- and CD8+ T-effector cell-mediated antitumor immune responses, representing thus, an excellent therapeutic weapon not only for symptomatic MM but also for the treatment of early disease, which may be highly sensitive to immune cell attack. Multiple Myeloma remains an incurable disease even with the use of modern therapies. Further progress made in the Tregs field in the context of MM could highlight their correlation with immune surveillance and disease outcome and enhance their implications in treatment strategies contributing to a new therapeutic paradigm.
